# Experimental Evidence of Persistent Androgen-Receptor-Dependency in Castration-Resistant Prostate Cancer

**DOI:** 10.3390/ijms140815615

**Published:** 2013-07-26

**Authors:** Takashi Kobayashi, Takahiro Inoue, Tomomi Kamba, Osamu Ogawa

**Affiliations:** Department of Urology, Kyoto University Graduate School of Medicine, 54 Shogoinkawahara-cho, Sakyo-ku, Kyoto 606-8507 Japan; E-Mails: selecao@kuhp.kyoto-u.ac.jp (T.K.); takahi@kuhp.kyoto-u.ac.jp (T.I.); kamba@kuhp.kyoto-u.ac.jp (T.K.)

**Keywords:** prostate cancer, castration resistant, androgen receptor, molecular target

## Abstract

In the majority of castration-resistant prostate cancer (CRPC), prostate-specific antigen (PSA), product of a gene that is almost exclusively regulated by the androgen receptor (AR), still acts as a serum marker reflecting disease burden, indicating that AR signaling is activated even under castrate level of serum androgen. Accumulated evidence shows that transcriptional ability of AR is activated both in ligand-dependent and -independent manners in CRPC cells. Some androgen-independent sublines derived from originally androgen-dependent LNCaP prostate cancer cells overexpress the AR and PSA, for which silencing the AR gene suppresses cellular proliferation. The overexpression of the AR confers androgen-independent growth ability on androgen-dependent prostate cancer cells. Some patient-derived prostate cancer xenograft lines also acquire castration-resistant growth ability secreting PSA. More recent publications have shown that the AR activated in CRPC cells regulates distinct gene sets from that in androgen-dependent status. This concept provides very important insights in the development of novel anti-prostate cancer drugs such as new generation anti-androgens and CYP17 inhibitors.

## 1. Clinical Aspects of Castration-Resistant Prostate Cancer

Although almost all prostate cancers initially respond to androgen-deprivation therapy (ADT) comprising medical or surgical castration, ADT is usually not permanently effective or curative, and most tumors eventually regrow after a certain period. Before docetaxel was recently proved to improve survival of prostate cancer patients after ADT failure [1,2], there had been no effective treatment left, and those patients are clinically categorized as a distinct disease stage. At this point, prostate cancers appear to have become a “hormone-refractory” disease characterized by “androgen-independent” tumor growth. Recent works have shown, however, that prostate cancers continue to depend on androgen receptor (AR) signaling despite low serum androgen levels. Consistently, serum prostate-specific antigen (PSA), a serine protease coded by a gene that is almost exclusively regulated by the AR, still plays a clinically important role as a serum marker reflecting disease burden. Therefore, prostate cancers that are progressing after castration are called castration-resistant prostate cancer (CRPC) [3,4]. Indeed, recent clinical trials have shown that a new generation anti-androgen [5] or novel inhibitor targeting androgen synthesis [6] is effective for post-docetaxel CRPC indicating that CRPC at that stage are still, at least in part, dependent on androgen/AR axis.

Recent works have accumulated direct and indirect evidence indicating that, in CRPC, AR signaling is activated even under the castrate level of serum androgen. This concept is very important since it means that AR itself and its downstream signaling can be still a therapeutic target in CRPC. However, molecular mechanisms for re-activation of AR in CRPC are complex and heterogeneous. Thus, it is indispensable to demystify detailed pathways for castration-resistant progression of prostate cancer. This review outlines recent advances in prostate cancer research and experimental evidence for the essential role of AR in CRPC.

## 2. AR Signaling Axis and Androgen Deprivation

In hormone-naive status, androgens circulate in blood in either a bound (to albumin or sex-hormone-binding protein) or free form. The majority (~95%) of circulating androgens is testosterone, produced by Leydig cells in testis under control of the pituitary hormone luteinizing hormone (LH). Free serum testosterone can transfer across plasma membrane into cytoplasm of prostatic epithelial cells, where unbound AR is abundant. Testosterone is then reduced by 5α-reductase into dihydrotestosterone (DHT), a more potent form of AR ligand. Upon binding of androgen, AR translocates to nucleus in a dimerized form and regulates gene expression by binding to specific hormone response elements.

Normal prostate epithelium and most of untreated prostate cancer cells are highly dependent on the androgen/AR axis and ablation or blockade of the signaling pathway results in severe regression of the tissue. Prostatic epithelial cells in culture stop proliferating at G1 cell-cycle phase (G1 arrest) upon androgen deprivation and then go to apoptosis. With the exception of rare small cell carcinomas of the prostate, most untreated human prostate cancers express AR at both the mRNA and protein level and respond to ADT. Physiologically, 95% of serum androgens are produced by testicular Leydig cells, while the rest are secreted by the adrenal gland. In ADT, surgical castration by bilateral orchiectomy, or medical castration by the administration of luteinizing hormone-releasing hormone analogue (either agonist or antagonist), suppress serum testosterone levels. Combined androgen blockade (CAB) comprises administration of an anti-androgen and surgical or medical castration. Castration suppresses serum testicular androgen levels and residual testicular and adrenal androgens are blocked by anti-androgens.

## 3. AR-Independent Pathways for CRPC

Molecular mechanisms for CRPC that progresses, by definition, despite castrate levels of serum testosterone can be categorized into roughly two concepts, namely AR-independent and -dependent pathways. AR-independent pathway, which is beyond the scope of this review, includes AR-independent activation of survival or anti-apoptotic pathway and mechanisms involving subpopulation of intrinsically AR-independent cancer cells. It has been known that androgen ablation induce activation of anti-apoptotic molecules such as BCL-2 [7], Clusterin [8], and AKT [9], which leads to the resistance by prostate cancer cells to castration. Some investigators reported that these pathways are promising therapeutic targets in CRPC [9–11].

The presence of a primarily AR-independent subpopulation in prostate cancer cells has long been suggested as a potential mechanism for castration-resistant progression of prostate cancer [12]. Although attractive, it has been only one theoretical model, and the presence of castration-resistant mouse prostatic epithelial cells has been documented recently [13]. Surprisingly, those are not basal cells as postulated initially but of luminal origin. It is still controversial which of luminal or basal epithelium is the cell of origin of prostatic adenocarcinoma, whether intrinsically castration-resistant luminal cells exist in human normal and malignant prostatic tissue, and whether the inherent castration-resistant subpopulation, if any, has a role in castration-resistant progression of prostate cancer.

## 4. AR-Dependent Pathways for CRPC

Thus, there is certainly an AR-independent subset of CRPC, however they are considered as a small minority, whereas the vast majority of CRPC maintain AR-mediated signaling activity and dependence on it. Reactivation of AR signaling in CRPC is clinically evident by re-elevation of PSA in most cases, and there is a response to secondary or tertiary hormonal therapy in the majority of them. Additionally, constant nuclear expression of AR in CRPC cells indicates that the AR signaling remains active despite the androgen withdrawal [14]. It is further supported by experimental findings that, in some models [15,16] as well as human surgical specimens [16–19], CRPC tumors express AR-targeted genes despite androgen ablation. Accumulated experimental evidence indicates that there are various mechanisms for the activation of an AR-mediated signaling axis under castrated conditions. They include *de novo* ectopic androgen synthesis and AR hypersensitivity or promiscuous activation caused by overexpression, mutation or truncation, interaction with co-factors, and posttranslational modifications. These discoveries have led to the development and introduction of novel therapeutic agents targeting the AR signaling axis toward clinical use.

## 5. *De Novo* Synthesis of Androgen (Figure 1A)

Expression profiles of castration-resistant tumors by Holzbeierlein and colleagues demonstrated that the gene expression changes detected in response to androgen ablation therapy were no longer present and that many of AR-driven genes were expressed, suggesting a reactivation of the androgen response pathway in the absence of exogenous androgen [15]. It was reported that castration-resistant bone marrow metastasis tumors [16] and circulating tumor cells from CRPC patients [17] express AR-regulated genes, indicating persistent activation of the AR signaling axis despite castration. Additionally, an increased expression of genes encoding steroidogenic enzymes was observed in these tissues and cells [16,17].

Locke and colleagues demonstrated intratumoral *de novo* synthesis of DHT in the LNCaP xenograft model for CRPC using liquid chromatography-radiometric/mass spectrometry (LC/MS) technique [18]. LNCaP tumors expressed all enzymes necessary to synthesize androgen and intratumoral androgens increased during CRPC progression in correlation to PSA upregulation. Montgomery and colleagues examined human hormone-naive and castration resistant metastatic prostate cancer tissue using LC/MS and found that the tissue testosterone level is higher in CRPC [19]. CRPC also displayed upregulation of genes encoding steroidogenic enzymes including CYP17A1, HSD3B1, HSD17B3, CYP19A1 and UGT2B17. These results clearly indicate that, despite anorchid serum testosterone levels, intratumoral steroidogenesis permits tumors to castration-resistant progression. These experimental findings drove researchers to develop inhibitors of androgenic enzymes and indeed abiraterone acetate, a CYP17 inhibitor, has been shown to improve survival of CRPC patients [6] and approved by the US Food and Drug Administration (FDA) for clinical use. Another CYP17 inhibitor, TAK700, is also currently under clinical trial [20].

## 6. AR Overexpression (Figure 1B)

One of the hallmarks of CRPC is overexpression of AR. A number of reports have shown increased expression of AR in human CRPC tissues based on gene expression [21–35] and immunohistochemistry [31,32] as well as circulating tumor cells from CRPC patients [17,36–38], although inconsistent results have been reported for a significant association between high AR expression and clinical outcomes [26,31,34]. High AR expression in CRPC is reproducible in some experimental models based on cell lines [39], cell line-based xenograft lines [40,41], and patient-derived tumor graft lines [42,43]. In a series from Erasmus Medical Center, Rotterdam, the Netherlands, the authors analyzed androgen dependent PC346 human prostate cancer cells expressing wild-type AR [39]. They further established sublines including those with acquired resistance to various androgen blockade therapies and found some of them overexpress AR and PSA. Chen and colleagues compared seven castration-resistant cell-based xenograft lines with their parental androgen sensitive lines and found that all seven tumors after castration-resistant progression showed modest increase in AR protein expression [40]. Our group has established sequentially transplantable xenograft lines from CRPC tissues that represent clinical features of prostate cancer including androgen sensitive regression upon castration followed by castration-resistant regrowth [42,43]. Notably, tumors in castration-resistant status overexpress AR protein. As a consequence of overexpression, increased AR sensitizes prostate cancer cells to low levels of androgen, leading to castration-resistant progression [40,44].

AR gene amplification partly accounts for the high expression of AR in some CRPC cases. Although AR plays an essential role in the carcinogenesis of prostate cancer, only a small minority of untreated prostate cancers harbor copy number alteration of AR gene [29,30,35,45,46]. In contrast, 10%–80% of CRPC cases have copy number gain of AR gene [23,24,26,27,31–33,35,37,45,46], indicating AR gene amplification is a driving force for castration-resistant progression of prostate cancer in those cases. Other possible explanations for AR overexpression include increased expression of AR gene without amplification (see an excellent review by Shiota *et al*. [47]) as well as posttranscriptional and posttranslational regulations of AR.

As for posttranscriptional modification, Yeap and colleagues demonstrated that AR mRNA stability is a major determinant of AR gene expression in prostate and breast cancer cells [48] and identified several RNA-binding proteins including HuA, CP1 and CP2 which bind to a highly conserved UC-rich region of 3′-UTR of AR mRNA and affect stabilization of AR mRNA [49]. Another group identified EBP1 as an RNA-binding protein that binds to the UC-rich region of the 3′-untranslated region (3′-UTR) of AR mRNA and stabilizes it [50].

Micro RNAs (miRNAs) modulate translation of mRNA by base-pairing interactions with 3′-UTR through destabilizing the mRNA or by repression of protein synthesis in actively translating ribosomes. However, association between miRNA-mediated regulation of AR and development of CRPC had not been evident until very recently [51]. A systematic analysis based on a gain-of-function screening of 1129 miRNAs by Ostling and colleagues identified 71 unique miRNAs that influenced the level of AR in human prostate cancer cells [52]. Of those, miR-34a and miR-34c expression was negatively associated with AR levels based on analysis of clinical prostate cancers. Another group has demonstrated that the overexpression of miR-488* downregulated the transcriptional activity of AR and inhibits the endogenous AR protein production in human prostate cancer cells [53]. Those results suggested that suppression of AR-targeting miRNAs may promote CRPC, although therapeutic potential of targeting these pathways is not fully understood.

AR is regulated also in posttranslational manners. It has been known that half-life of AR protein is prolonged in the presence of agonistic steroids including testosterone, dihydrotestosterone, R1881 (synthetic androgen), and other nonandrogenic steroids in prostate cancer cells [54,55] and others [56–60]. Effectiveness of stabilization is correlated with affinity of ligands to AR and abolished by AR antagonists including hydroxyflutamide and bicalutamide [57,59]. Stabilization of AR increases abundance of AR protein and usually promotes androgen-dependent proliferation. However, it was reported that AR needs to be degraded during mitosis for subsequent cell-cycle progression and too much stabilization of AR inhibits mitotic AR degradation and next cycle of cell division [55]. This can explain biphasic effect of androgen on the cell-cycle progression of androgen-dependent prostate cancer cells [61]. Importantly, androgen-independent prostate cancer cells showed a prolonged half-life of AR in the absence of androgen [54], indicating that AR stabilization has a potential role in AR overexpression in CRPC.

More recently, some molecules that are responsible for AR stabilization have been identified. Thomas and colleagues demonstrated that Stat5 (signal transducer and activator of transcription 5) stabilizes AR and potentiate castration-resistant progression of prostate cancer cells [62]. They also found Stat5 expression is increased in high-grade disease or CRPC tissues. Cyclin-dependent kinases (Cdk) 1 [63] and 5 [64] were also reported to bind to and stabilize AR. Interestingly, Cdk5 stabilizes AR through phosphorylation of AR at Ser-81 [64], whereas Cdk1 does not require phosphorylation at Ser-81 for AR stabilization although it phosphorylates AR at Ser-81 [63]. Future studies will discover more molecules as a posttranslational modifier of AR, and elucidate their detailed roles *in vivo* for castration-resistant progression and identify potential targets in CRPC patients.

## 7. Non-Specific Ligand Recognition (Figure 1C)

AR sometimes recognizes steroidal or non-steroidal ligands other than testosterone or dihydrotestosterone like an agonist, which leads to aberrant activation of AR in the absence of androgen. Clinicians often observe apparently a paradoxical PSA decrease upon discontinuation of first generation non-steroidal anti-androgens including flutamide and bicalutamide, the so called “anti-androgen withdrawal syndrome (AWS)” first described by Kelly and Scher [65]. This phenomenon indicates that anti-androgens sometimes stimulate prostate cancer cells for PSA production, and probably AR-mediated proliferation, causing selection pressure that induces prostate cancer that can grow on anti-androgen. Veldscholte and colleagues discovered that androgen-dependent human prostate cancer LNCaP cells harbor the AR with single missense mutation on ligand-binding lesions (Thr877Ala) [66]. They demonstrated that androgens, progestagens, estrogens and anti-androgens bind the mutated androgen receptor protein and activate the expression of an androgen-regulated reporter gene.

Hara and colleagues established a bicalutamide-resistant subline from LNCaP cells and found the cells harbor Trp741Cys/Leu mutation in the AR gene, which recognized bicalutamide as an agonist [67]. Our group established a patient-derived tumor graft line that expressed AR Trp741Cys that recapitulated AWS and effectiveness of alternative anti-androgen therapy *in vivo* [42,68]. Indeed, it was reported that 10%–30% of prostate cancers treated by anti-androgens acquired point mutation in the AR gene [69–72]. Thus, it has been believed that mutant AR is responsible for the “promiscuous” ligand recognition toward CRPC progression particularly under use of anti-androgens [73]. However, Chen and colleagues demonstrated that modest overexpression of AR also results in less specific ligand recognition on prostate cancer [40], indicating AWS can be expected in more cases with disease progression against anti-androgen administration.

## 8. Ligand-Independent AR Activation (Figure 1D)

Some clinical and experimental evidence indicate that there are several mechanisms that confer AR activation to prostate cancer cells relatively or completely independent (hereafter referred to as simply “ligand-independent”) of activating ligands. There are a number of co-factors for AR (extensively reviewed in ref. [74–76]), and some of them are overexpressed in CRPC and activate transcription by AR [77]. Recently, forkhead box protein A1 (FOXA1) has been shown to play an important role as AR co-regulator in prostate cancer. FOXA1 primarily an enhancer pioneer transcription factor and acts as a global mediator of steroid receptor action in hormone-dependent cancers including prostate cancer [78]. It was reported FOXA1 gene was amplified [79] and overexpressed [80,81] in metastatic prostate cancers and particularly in CRPC cases. Recent reports demonstrated that FOXA1 acted as an AR cofactor to drive G2/M cell-cycle progression [82] and that AR and FOXA1 functionally cooperate and enhance proliferation and migration of prostate cancer cells [80] although there are conflicting reports on FOXA1 function in metastatic progression of prostate cancer [80,83].

Additionally, FOXA1 was shown to regulate a large number of genes considered to be AR-pathway-specific through binding and activating the glucocorticoid receptor [84]. In that context, activation of glucocorticoid receptor may promote CRPC progression whereas corticosteroids are used for CRPC patients to suppress adrenal androgens via an endocrinological negative feedback loop. Moreover, Zhang and colleagues showed that FOXA1 promote G1/S cell-cycle progression in an AR-independent manner [85]. According to a recent report by Jin and colleagues, FOXA1 requires the AR pathway to induce cell growth whereas it inhibits cell motility and epithelial-to-mesenchymal transition (EMT) in an AR-independent manner [83]. Another report showed a mutually inhibitory cross-talk between FOXA1 and SLUG1, a master regulator of EMT [86]. Recently, recurrent mutations in FOXA1 were observed on residues in the forkhead domain that resides near the DNA-binding surface [87] and the mutated FOXA1 was reported to increase tumor growth [88]. Thus, FOXA1 plays important roles in CRPC progression through co-regulating AR activity and distinct functions, however the role of FOXA1 in CRPC remains inconclusive and will be determined in the future.

Posttranslational modifications including phosphorylation, acetylation, sumoylation, ubiquitination and methylation that contribute to ligand-independent activation of AR transcriptional activity have been widely studied [89,90]. Among those, phosphorylation is most extensively studied. Culig and colleagues demonstrated that, in human prostate cancer cells, various growth factors including insulin-like growth factor 1 (IGF-I), keratinocyte growth factor (KGF), and epidermal growth factor (EGF) directly activate the AR in the absence of androgens [91]. Indeed, Craft and colleagues found that castration-resistant xenograft sublines of human prostate cancer cells expressed higher levels of the HER-2/neu, receptor tyrosine kinase activated by EGF [92]. They also showed that overexpression of the HER-2/neu conferred castration-resistance to LAPC-4 and LNCaP cells via AR activation. As a downstream effector of HER-2/neu, MAP kinase was shown to upregulate AR transactivation through phosphorylation of AR at serine 514 resulting in the promotion of interaction between AR and AR coactivators, such as ARA70. Consistent with this, a siRNA phenotypic screen identified MAK3K11 as a kinase regulating prostate cancer cell growth through modulating the AR transcriptional program [93]. Protein kinase A (PKA) is also known to phosphorylate and activate AR [94]. Our group showed that using a patient-derived tumor graft model, a prostaglandin E2 receptor EP4 conferred castration-resistance via activating PKA-AR signaling [43].

In addition to serine/threonine kinases, involvement of some tyrosine kinases has been suggested for castration-resistant activation of AR. Mahajan and colleagues found that activated Cdc42-associated tyrosine kinase (Ack1) phosphorylated AR protein at Tyr-267 and Tyr-363 and promoted castration-resistant growth of LNCaP and LAPC-4 xenograft tumors [95]. Guo and colleagues reported that Src tyrosine kinase phosphorylated AR and conferred castration resistance to prostate cancer cells upon EGF stimulation [96]. The same group demonstrated that overexpression of non-receptor tyrosine kinase Etk/BMX induced AR phosphorylation and castration resistance, suggesting that Etk/BMX is another tyrosine kinase of AR associated with castration-resistant progression of prostate cancer [97]. AR has been reported to bind to and activate Src in a ligand-dependent but non-transcriptional manner [98] and Src activation has been reported to activate other signaling pathways that confer androgen independence to LNCaP cells without affecting AR transactivation [99]. Thus, interaction between Src tyrosine kinase and AR seems complex and will be elucidated in the future studies.

The PI3K/AKT pathway also plays complicated roles both in castration-naive and -resistant prostate cancers. Reports have shown that androgen withdrawal results in an increase of PI3K/AKT pathway activity both in cultured cells [100,101] and *in vivo* [102,103], which supports survival after androgen ablation. Although Wen and colleagues reported that activation of AKT upregulated AR transactivity [104], there have been conflicting reports that activation of AKT suppressed AR transactivity [102,103,105]. Additionally, activation of mTOR pathway, a main downstream effector of the PI3K/AKT pathway, was also reported to have oncogenic functions both in castration-naive and -resistant prostate cancers [106–108]. Cinar and colleagues reported that genetic or pharmacological downregulation of mTOR resulted in an increased abundance of AR protein, which is regulated by mRNA translation initiation [109]. Collectively, PI3K/AKT/mTOR pathway seems to contribute prostate cancer progression not through activation of AR but by facilitating survival of prostate cancer cells upon androgen deprivation. As another siRNA screen revealed, concomitant targeting of this pathway along with AR targeting therapy is a potentially effective strategy to eradicate prostate cancer [110].

Truncated AR species that composes only *N*-terminal domain (NTD) and DNA-binding domain (DBD) but lacks ligand-binding domain (LBD) and *C*-terminal domain (CTD) have been recently discovered as novel molecular mechanisms for the castration-resistant progression of prostate cancer. It was already known that 22Rv1 prostate cancer cells expressed smaller 75–80 kDa AR species that are distinct from wild-type ~112 kDa AR protein [111]. Antibody mapping revealed that the smaller AR lacks LBD and CTD. By a recent series of efforts based on 3′-rapid amplification of cDNA ends (3′-RACE), it was demonstrated that these AR truncates were coded by splice variants of AR mRNA harboring cryptic exons [112–115]. After some more additional isoforms were identified in VCaP cells by deep sequencing combined with 3′-RACE [116], more than 10 variants for truncated AR are known to date [117].

Indeed, the AR truncated variants are frequently found in CRPC tissues [118,119] and induced by androgen deprivation in prostate cancer cells [120]. Intragenic rearrangement of an AR genomic segment was proposed as a pathologic origin of the emergence and expansion of a subpopulation expressing high levels of truncated AR isoforms during CRPC progression [121,122]. Another group found that expression of truncated variants increased acutely in response to androgen withdrawal, indicating that the increase in AR variants expression in CRPC is an acute response to castration rather than clonal expansion of castration, or antiandrogen-resistant cells expressing gain of function AR variants [116]. In theory, these AR variants lacking LBD function as constitutively active in a ligand-independent manner, and prostate cancer cells expressing the AR variants are resistant to ADT including CYP17A inhibitors [117,123]. It was also reported that prostate cancer cells expressing the AR variants were resistant to the second-generation antiandrogen MDV3100 [124], whereas another study showed that castration-resistant growth mediated by the AR variants was blocked by MDV3100 or by selective siRNA silencing of full-length AR, indicating that some AR truncated variants require full length AR to drive castration-resistant progression of prostate cancer [116].

## 9. Targeting AR in CRPC; Promise and Peril

Thus, AR drives CRPC progression in various fashions. Indeed, recent advances in transcriptome and genome revealed that AR is one of the most frequently altered genes in prostate cancer [88]. Moreover, AR mediates distinct transcription programs in CRPC from that in castration-naive prostate cancers. Wang and colleagues conducted chromatin immunoprecipitation (ChIP)-based screen for AR-dependent target genes in androgen-dependent and -independent prostate cancer cells and found that AR selectively upregulated M-phase cell-cycle genes in androgen-independent cells, including UBE2C, a gene that inactivates the M-phase checkpoint [82]. Similar study by another group also showed a distinct AR target gene set in CRPC cells [125]. Hu and colleagues showed that AR truncate variants mediates distinct transcription program from that mediated by ligand-dependent full-length AR [118]. Sharma and colleagues analyzed AR binding sites in human CRPC tissues and xenograft tumors and identified a number of AR binding sites, most of which were not identified in cell lines [126]. These data collectively indicates that AR is persistently functioning even under castrated condition but differentially regulating cellular function. It is also indicated that AR target genes are highly context-dependent and that it appears to be difficult to identify a single or a few genes that can be universally targetable elements in heterogeneous tumors. Therefore, it looks reasonable to target AR as a master regulator of androgen-dependent and -independent prostate cancer growth.

With a castration-resistant status, prostate tumor models showed that AR is still required for growth. Chen and colleagues knocked down AR using short-hairpin RNA (shRNA)-expressing lentivirus in castration-resistant subline of LAPC-4 and LNCaP cells and showed the growth of xenograft tumors from both cells were retarded compared to control infected with control shRNA-expressing virus [40]. Another group used doxycycline-inducible shRNA system and observed similar findings on LNCaP cells [127]. Zhang and colleagues used antisense oligonucleotide that was shown to specifically downmodulate AR mRNA and protein and observed growth repression in a castration-resistant C4-2b xenograft model [128]. Since Chen and colleagues have shown the castration-resistant activation of AR required DNA-binding and nuclear translocation [40], the second generation antiandrogens are multitargeting; nuclear translocation, DNA-binding, recruitment of RNA Pol II as well as ligand binding [129,130].

Another effort is to target NTD that can theoretically and indeed cover AR truncate variants as a target [131]. NTD contains AF-1 region that plays an essential role for active transcription through interactions with other proteins including bridging factors and the basal transcriptional machinery. Currently three classes of these agents, small molecule [132], peptides [133], and decoy DNA [134], have been shown to suppress tumor growth in preclinical studies using xenograft models. As described above, using a gain-of-function miRNA screen and functional validation by Ostling and colleagues identified unique miRNAs that bound to 3′-UTR of AR mRNA and attenuated its expression [52]. This is also a potential strategy to inhibit AR function in CRPC.

Some castration-resistant cell lines express little AR compared to their androgen-dependent parental lines [39,61] as well as primarily androgen-independent cell lines such as PC3 and DU145. In those cell lines, aberrant methylation of *AR* promoter is suggested as an associated molecular mechanism [135]. It was also reported that some CRPC patient-derived tumor grafts harbor features like small cell carcinoma and do not express AR [136]. In these models, some signaling pathways that are regulated by AR in androgen-dependent cells seem to be aberrantly activated in an AR-independent manner [61,99]. Therefore, although there is no doubt AR plays a substantial role in the majority of CRPC, it should be noted that some CRPCs bypass the androgen/AR axis and have a proliferation signaling pathway activated.

## 10. Outlooks

AR drives CRPC even under castrated serum androgen level. There are a variety of mechanisms for the castration-resistant activation of AR and the AR in CRPC regulates distinct gene sets from that in androgen-dependent prostate cancer. These indicate that AR persistently stays at the center of therapeutic targets even after castration-resistant progression of prostate cancer.

## Figures and Tables

**Figure 1:**
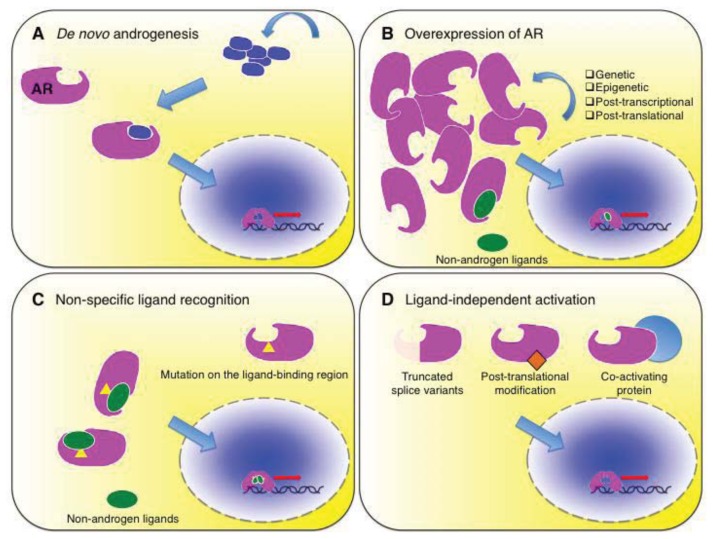
Schematic mechanisms for castration-resistant activation of the androgen receptor (AR). (**A**) *De novo* androgen synthesis results sustained levels of intratumoral androgens; (**B**) Genetic, epigenetic, post-transcriptional, or post-translational aberration that increases abundance of AR leads to ligand-independent activation or non-specific ligand recognition; (**C**) A mutation on the ligand-binding region also causes non-specific ligand recognition; (**D**) Truncated splice variants lacking the ligand-binding region, post-translational modification such as serine/threonine or tyrosine phosphorylation, or interaction with co-activating factors activates AR in a ligand-independent manner.
